# Identifying an APP-binding protein in neuronal cell death

**DOI:** 10.1186/1750-1326-7-S1-L22

**Published:** 2012-02-07

**Authors:** Yunwu Zhang, Huaxi Xu

**Affiliations:** 1 Fujian Provincial Key Laboratory of Neurodegenerative Disease and Aging Research, College of Medicine, Xiamen University, Xiamen, Fujian 361005, China; 2 Neurodegenerative Disease Research Program, Sanford-Burnham Medical Research Institute, La Jolla, California, USA

## Background

Apoptosis is an essential cellular process involved in multiple diseases and a major pathway for neuronal death in neurodegeneration. The detailed signaling events/pathways that lead to apoptosis, especially in neurons, require further elucidation.

## Results

Here we find that a mitochondrial solute carrier family protein, appoptosin, induces reactive oxygen species release and intrinsic caspase-dependent apoptosis. The physiological function of appoptosin is to transport/exchange glycine/5-amino-levulinic acid across the mitochondrial membrane for heme synthesis. Alzheimer’s β-amyloid precursor protein interacts with appoptosin and modulates appoptosin-induced apoptosis. Levels of appoptosin are upregulated in brain samples from Alzheimer’s disease and infarct patients and in rodent stroke models, as well as in cells treated with β-amyloid (Aβ) and glutamate. Downregulation of appoptosin prevents the cell death and caspase activation caused by glutamate or Aβ insults.

## Conclusion

Our study identifies appoptosin as a crucial player in apoptosis and a novel proapoptotic protein involved in neuronal cell death, providing a possible new therapeutic target for neurodegenerative disorders and cancers.

